# Resolutions of the Albany County Medical Society Relative to Abortion

**Published:** 1871-11

**Authors:** 


### Resolutions of the Albany County Medical Society Relative to Abortion.

Dr. T. D. Crothers offered the following resolutions, which were unanimously adopted :—

*Whereas,* The subject of Criminal Abortion is agitating the public mind, by the late astonishing disclosures of its prevalence, and that in our vicinity, as elsewhere, unscrupulous specimens of human depravity, professional abortionists, are plying their fiendish traffic, and from the members of the medical profession is expected a public expression of their position in regard to this widespread evil, therefore

*Resolved,* That we recognize the procuring of abortion as criminal in the highest degree, unless absolutely demanded for the purpose of saving human life.

*Resolved,* That, as physicians seeking to obviate the cause of many intractible diseases which are met with every day—diseases which destroy the physical powers and undermine the foundation of truth, morality, and happiness of society—we pledge all our efforts to support any legislation, or other measures, which our law officers may propose, and which promises to mitigate, if not remove, this infamous practice and the fearful consequences flowing from it.

*Resolved,* That we are bound, by a sense of duty as citizens and physicians, to condemn the abortionist, his associates and abettors, and deem them unworthy of our association, respect, or notice, using all means in our power to expose their nefarious schemes and bring them to punishment.

*Resolved,* That we not only denounce this traffic in human life, but try to infuse into the public mind a proper understanding of the fearful and inevitable punishment which follows this violation of the physical laws, believing that, with the aid of an intelligent people, this reform, so much needed, would be rapidly brought about.

*Resolved,* That a copy of this preamble and resolutions be forwarded to each of the daily papers of this city, with a request for gratuitous publication; that the position of the Albany County Medical Society be, beyond all doubt in this important matter, and to secure similar expressions from other societies and the profession generally throughout the State.

